# Expanding the Focus on Work Factors in an Outpatient Setting: Does a Nudge of Patients and Clinicians have an Effect on Return-to-Work and Benefits? Findings from the NSAC Nudge Multicentre Randomised Controlled Trial

**DOI:** 10.1007/s10926-025-10343-x

**Published:** 2025-12-12

**Authors:** Ingvild Bardal, Nils Abel Prestegård Aars, Laurent Olivier Trichet, Oda Lekve Brandseth, Christoffer Terjesen, Eirik Irgens, Bernt Ole Hansen, Aina Kristoffersen, Anje Höper, Odd Rune Jenssen, Beate Brinchmann, Arnstein Mykletun

**Affiliations:** 1 Centre for Work and Mental Health, Nordlandssykehuset HF, Bodø, Norway; 2 Department of Community Medicine, UiT, The Arctic University of Norway, Tromsø, Norway; 3 HelseIArbeid, Nordland Hospital Trust, Bodø, Norway; 4 HelseIArbeid, University Hospital of Northern Norway, Tromsø, Norway; 5 HelseIArbeid, Helsepartner Rehabilitering, Alta, Norway; 6 HelseIArbeid, Finnmark Hospital Trust, Kirkenes, Norway; 7 Department of Occupational and Environmental Medicine, University Hospital of North Norway, Tromsø, Norway; 8 HelseIArbeid, Helgeland Hospital Trust, Sandnessjøen, Norway

**Keywords:** Sickness absence, Return- to- work, Work factors, Musculoskeletal disorders, Mental health

## Abstract

**Purpose:**

The purpose of the study was to test the effects of a nudge on patients return-to-work (RTW) and benefits in a clinical service aimed to promote RTW and prevent sickness absence in patients referred for musculoskeletal disorders or mental illness.

**Methods:**

The study was a multicentre, single-blinded, parallel two-arm randomised controlled trial design. The setting was five outpatient clinics in Northern Norway, offering a work-focussed treatment to patients referred for common mental- and/or musculoskeletal disorders. Patients were randomised to receive either a questionnaire mapping health factors only, or a questionnaire mapping also Motivation for work, Barriers for return-to-work and Work environment (MBW). Clinicians were also nudged by receiving patient reports based on the patient survey. Data on sickness absence and benefits was retrieved from national registries after one year of follow-up.

**Results:**

Among 1171 patients consenting to participate in the study, 1091 were eligible for inclusion in the main analyses. The intervention group (n = 558) did not differ significantly from the control group (n = 533) with respect to key variables at baseline. The nudge had no effect on days of sickness absence, return to work rates, or disability benefits during follow-up.

**Conclusion:**

This attempt to nudge patients and clinicians on MBW factors did not influence RTW or benefits during one year of follow-up. Although MBW factors were prevalent at baseline, the results suggest that increasing work focus in a setting where clinicians already address these factors to some extent has little effect.

**Trial Registration:**

The trial was prospectively registered at clinicaltrials.gov on August 9th, 2021. Trial identifier NCT 05006976 (https://clinicaltrials.gov/study/NCT05006976). In this study we report on the primary outcome listed as “Functional recovery: sickness absence registry data”.

**Supplementary Information:**

The online version contains supplementary material available at https://doi.org/10.1007/s10926-025-10343-x.

## Introduction

In many welfare states, employees have the right to sickness absence (SA) benefits when ill, providing essential support. However, SA can also have unintended negative consequences for both individuals and society: those unable to work often experience worsened health and quality of life [1], there is evidence for work having a protective effect on mental health [2], and return to work (RTW) is emphasised as a treatment goal both for patients with musculoskeletal- and mental disorders [3–5]. From a societal perspective, high- and increasing rates of SA and disability lead to a loss in productivity and increasing public expenses [6], and thus pose a challenge to the sustainability of welfare systems in many Western countries. Much of the SA and reduced work capacity are attributed to common mental disorders (CMD) and musculoskeletal disorders (MSD), conditions that are highly prevalent and have a high demand for effective interventions [7–10]. This has implications for capacity and resource utilisation within the healthcare system. Consequently, policies and interventions aimed at reducing SA and disability pensions (DP), as well as increasing employment, are of considerable focus in OECD countries [7].

In accordance with the biopsychosocial model, it is well established that SA is influenced by more than just medical conditions alone; several factors, including social determinants, can also contribute [11, 12]. For instance, treating the symptoms of mental or musculoskeletal disorders alone may be insufficient to reduce SA [13]. At the patient level there has been an increase in work-focussed clinical interventions and studies aimed at reducing SA, and it has been suggested that integrating a focus on RTW strategies within health interventions is of importance for RTW [4]. However, research has struggled to demonstrate consistent long-term benefits of work-related interventions on RTW or SA status and there have been uncertainties regarding the effect on RTW of work-related interventions for patients with different diagnoses [14, 15]. In two recent systematic review and meta-analysis, no particular work-related intervention was found to be more efficient than others for RTW [16], but including variants of cognitive behavioural therapy (CBT) interventions, including work-focussed CBT, showed promising results for RTW outcomes for both CMD and MSD [17]. In another systematic review, the authors did not reach a conclusion regarding which elements make vocational rehabilitation interventions effective (or ineffective) in facilitating return to work among people on long-term SA. Nonetheless, they identified several features commonly associated with successful interventions, including counselling, motivational interviewing, return-to-work planning, work placement, practical skills training, and workplace advice [18]. In summary, addressing work-related factors seems to hold promise for improving RTW outcomes. There are several mechanisms which can explain why a work-focussed approach may be useful.

First; symptoms of mental disorders and musculoskeletal pain among working-age populations and the need for SA may originate from workplace environmental factors, work-family conflict, or general life stressors [19–23]. These factors do not always surface during clinical situations. Failure to acknowledge or investigate the underlying cause of symptoms may carry the risk of medicalisation, promotion of sick roles and increased healthcare use. Second; self-perceived barriers for RTW can challenge RTW after long-term SA [24]. Barriers for RTW may be relational, cognitive, or emotional and could be caused by- or worsened- during SA. These barriers may prolong the RTW and exacerbate health problems [24]. Third; the patient’s motivation plays a role in the decisions about work or SA. Motivation is influenced by complex structures of beliefs, attitudes, social norms, and personal goals [25] and social learning theory provides a framework for understanding how motivation influences the process of RTW [26, 27]. Following prolonged SA, RTW can be viewed as behavioural change process, in which self-efficacy beliefs, goals, outcome expectations and perceived environmental factors and barriers together shape motivation and behaviour [26], whose potential modifiable nature makes it a target for interventions [28, 29]. This suggests that addressing motivation in clinical care has potential to influence RTW trajectories.

The implication is that these three factors represent a potential for improving clinical care for patients with common mental- and/or musculoskeletal disorders who are on- or at risk of SA. Increasing the focus of both the patient and the patient’s clinician on these factors thus holds a potential for both improving RTW and symptom alleviation. Such a focus may be facilitated by a “nudge”. This concept refers to any aspect of the choice architecture that alters people’s behaviour in a predictable way without restricting their options, and builds on a psychological theory on how people make decisions [30]. However, a systematic review and meta-synthesis states that there is no clear technical definition of the term nudge, and efforts to categorise nudging have led to various models. Numerous techniques have been described, making the distinction between nudges and non-nudges unclear [31]. Nudges has been applied in a variety of settings for an array of different outcomes and purposes [32], including public health [31, 33, 34], patients in a healthcare setting [35], and clinicians [36–38] and within the field of health economics [39]. Nudges have also been successfully applied for RTW outcomes, for example by introducing compulsory dialogue meetings between employers, the sick-listed employee, and the employee’s general practitioner [40]. Although nudging has gained popularity within health domains, the effect size of nudges varies considerably across studies and some nudges have been proven to be ineffective [31].

In this RCT we are testing whether a nudge promoting focus on Motivation for work, Barriers for RTW and Work environment (MBW) has an effect on work outcomes or benefits in a work-focussed outpatient clinic for patients with CMD and/or MSD. The intervention is a pre-consultation questionnaire covering MBW factors distributed to patients and includes the provision of a summary of the patient’s response to physiotherapists, psychologists, and medical doctors at the clinic prior to consultation. We are not aware of any previous studies testing this particular form of nudging for RTW improvement in a specialised work-focussed setting.

The anticipated mechanism of the nudge intervention was that the questionnaire would lead to 1) an increased expectation among patients that MBW factors will be addressed during treatment, and/or 2) an increased focus on MBW factors by the clinician during treatment, both due to the report and the patient’s expectations. We thus hypothesised that the nudge would frame the clinical dialogue concerning MBW factors, and that this in sum would have a positive effect on work outcomes.

## Methods

The setting, study population, data collection, and the intervention in the Norwegian Sickness Absence Clinic Nudge (NSAC Nudge) trial is described in greater detail in a peer reviewed and published protocol [41]. The following provides a concise description of details relating to data collection, setting, and design and we refer readers to the protocol and the trial registration [42] for a comprehensive account of the study details.

## Study Design, Setting, and Participants

The study is a multicentre randomised controlled trial (RCT), which recruited patients from five [*HelseIArbeid]* (HIA) outpatient clinics in Northern Norway. The HIA clinics offer coordinated, multidisciplinary outpatient services to patients with MSD and/or CMD (mild to moderate anxiety or depression) who wish to remain in, or return to, work or education. The clinics constitute the specialist health care sectors designated service for this patient group and the study was set to these clinics because of a scientific- and clinical interest to investigate whether a focus on work factors could be further enhanced in this setting.

The HIA treatment approach is work-focussed and provided by interdisciplinary teams which typically include medical doctors specialised in physical medicine and rehabilitation, psychologists, physiotherapists and employment support supervisors from the Norwegian Labour and Welfare Administration (NAV). Treatment is tailored to individual needs and may include a combination of monodisciplinary, interdisciplinary, and interagency consultations. Clinicians provide guidance and support, tailored at the individual, including information about the possible causes of ailments and treatment options such as therapy, specific exercise programmes, physical activity, medication, activity plans, and self-help strategies. The consultations may consist of CBT for light-to moderate anxiety-, depression- or fear avoidance, brief intervention for debilitating musculoskeletal pain, CBT-I for sleep disorders or information and counselling. It can also involve work adaption, and available support from NAV, including rights, responsibilities, and career guidance. There is a focus on return to work and the benefits of remaining in work despite loss of function throughout [43].

Patients are referred to HIA by general practitioners (GPs) and eligibility for treatment is assessed by a team of clinicians, based on national guidelines for diagnosis and symptom severity.

## Recruitment

Upon referral to HIA, a research coordinator (RC) at each outpatient clinic contacted the patient via telephone, informed about the study and retrieved the patient’s email address. The RC then entered the patient’s details on an online custom-built interface linked to a digital survey tool (Tivian A/S, Oslo Norway). An automated email containing further details and a consent form, but not an actual question for consent, was immediately sent to the patient. After 24 h, a new automated email was sent, this time including a link to the survey. On the first page of the survey, the patient was asked to consent to participate. The survey tool handled randomisation immediately and blinded to the patient. Depending on randomisation result, the patient was then forwarded to either the survey of the intervention group or the control group. This procedure took place approximately two weeks prior to first consultation at the clinic. In case the patient chose not to participate, the survey containing health questions only was provided for clinical purposes.

## Inclusion and Exclusion Criteria

Patients were eligible for inclusion in the trial if they were offered a consultation at one of the HIA clinics and were ≥ 23 years of age at time of referral. The age limit was set on the basis of national guidelines on deadlines for assessment according to different diagnoses, which have particular cut-offs for patients referred on the basis of mental health conditions such as depression and which are aged < 23 years [44]. This exclusion criteria was applied to comply with the exclusion criteria in another trial conducted in the same region at a later time point [45], thus allowing for comparability between the samples.

**Fig. 1:**
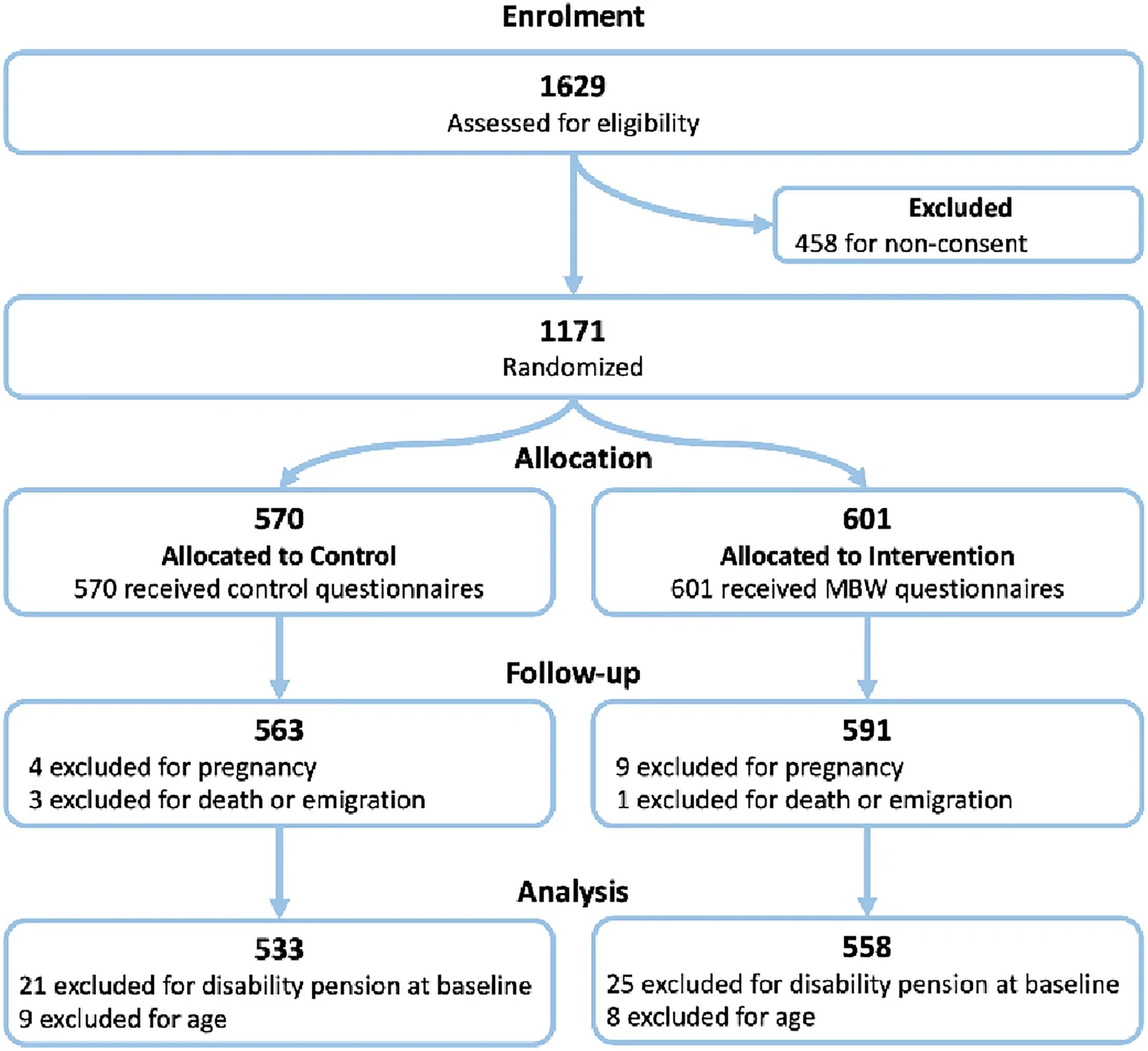
Flow chart for enrolment, exclusion, randomisation, allocation, follow-up, and analysis of participants in the study

A total of 1171 patients were included from five different HIA clinics in Northern Norway over a period of 16 months, from 06.09.2021 to 12.01.2023. The inclusion period ended when the sufficient sample size had been confirmed. For the analysis of the main outcome, we excluded patients receiving DP at baseline or patients which received pregnancy-related benefits, emigrated or deceased during the follow-up period or were aged ≥ 62 years at baseline. Our final sample included 1091 patients, where 558 were randomised to the intervention group and 533 were randomised to the control group. Figure 1 presents a flow chart for inclusion and exclusion.

## Intervention

Patients were randomised in equal proportions to receive either of two questionnaires prior to consultation at a HIA clinic: 1) health survey only (control group) or 2) health survey *and* survey on patient’s MBW (intervention group, nudge). The survey aimed to cover topics of importance for SA. To tailor the survey to the individual patient’s need we used indexes to regulate the detail of each topic. Patients who reported values below the pre-specified value on any index question did not receive further questions on the topic concerned. These pre-specified cut-offs were determined based on published psychometric properties of the different questionnaires included where available [41], or upon discussions within the research group.

For both randomisation groups, the patient’s clinician received a summary report of the patient’s responses prior to consultation. This report highlighted potential areas of concern using colour codes and summary scores. The colour codes indicated either “area of concern” (orange), “may be of concern” (yellow), or “not of concern” (green). The clinicians were not restricted to treating only patients from the intervention- or the control group, and we made no attempt to blind clinicians of randomisation result. Since the HIA clinics already had a work-focus, the clinicians were not prohibited from exploring MBW factors themselves during consultation. A focus on work factors in the clinic can thus be considered part of treatment as usual. Prior to the study, and also for patients in the control group, information on MBW factors would typically be obtained orally during patient consultations or in shorter questionnaires, but less comprehensive and less structured than in the intervention. Colour coding for the individual instruments were set before the study was initiated and based on established cut-offs where available or after discussions with a team of experienced clinicians from the HIA clinics.

## Outcome

The outcome in the study was work and benefits, operationalised as days on DP, work assessment allowance (WAA), full sickness absence (FSA), and graded sickness absence (GSA) in the 12-month period following recruitment for each individual. These data were obtained from NAV and linked to the patient’s questionnaires using the unique personal identifier. For days where no benefits were recorded, patients were categorised as “other”. This category includes employment, education, and unemployment. Using incomplete data from NAV on contracted work hours, we performed sensitivity analyses which confirmed most days in the category “other” was indeed employment. Since NAV only records SA exceeding 16 days, short-term absences were not registered in our material.

## Baseline Variables

In the NSAC Nudge the questionnaire and the summary report itself constituted the intervention, and accordingly only the intervention group was given the opportunity to respond to the entire questionnaire. In Table 1 we present baseline characteristics from the patient’s response to the baseline survey, with the exception of work/SA status which was retrieved from NAV data.

For general demographic characteristics we included age, gender, level of education and whether patients worked in the public or private sector. For work/SA status we present the proportion of patients on GSA, FSA, WAA, or DP at time of inclusion, as well as length of current SA spell for those patients sick listed at baseline. For self-reported health status, we present the patients extent of musculoskeletal problems and their levels of anxiety and depression, the latter measured using the Hopkins Symptom Checklist 10 (HSCL10). A score at 1.85 or higher is estimated to represent case level symptoms of anxiety and depression [46].

Motivation was assessed by asking patients about their beliefs and wishes relating to work or benefits during follow-up on a scale from 1 (do not wish/believe) to 5 (do wish/believe). In Table 2 we include the patients mean score for both beliefs and wishes regarding work/benefits in 12 and 24 months, respectively.

Barriers for RTW were assessed by asking patients on SA to state their level of agreement (scale from 1 (do not agree) to 5 (agree)) to a total of 26 statements regarding beliefs about potential barriers for return to work [41]. We included the proportion of patients with a maximum score (5) on any of the items.

We included patient responses to questions used as index for established work-environment or work-life balance questionnaires. This includes effort-reward imbalance (ERI: scored from 1 “strongly disagree” to 4 “strongly agree”) [47], demands, control and social support (DCS: demand and control are scored from 1 “No, almost never” to 4 “Yes, often” and social support is scored from 1 “strongly disagree” to 4 “strongly agree”) [48] and work-home interference (WHI: scored from 1 “never” to 4 “almost always”) [49]. Further, we present the reported prevalence of bullying at the workplace, measured using the Short negative acts questionnaire [50], and whether the employee is exposed to noise, pollution, chemicals, or unfavourable working positions.

Prior to analyses we checked whether the intervention and the control group differed with respect to gender, age, SA status and symptom severity, and observed no significant differences. A complete list of the questionnaires and data sources can be found in the protocol paper [41] and in the trial registration [42].

## Power Calculation and Statistical Analysis

Using a beta level of 0.2, an alpha level of 0.05, and an assumed difference in effect size of 10%, power calculations indicated that the total number of participants needed for the study was > 1100 [41].

Descriptive statistics were used for baseline characteristics. The effect of the intervention on the outcomes was assessed using an independent sample t-test, reporting the two-tailed significance. Since the distribution of SA is often skewed, we also performed non-parametric test (Mann–Whitney U test) of differences in outcome between the groups. The significance level was set at p < 0.05. We also present cross-sectional distribution of days per month of each outcome for the intervention- and control group separately. Data management and linkage of data sources were performed using Python version 3.8 (Python Software Foundation) and IBM SPSS Statistics for Windows, Version 29.0 (IBM Corp. Armonk, NY). Statistical analysis was conducted using Stata MP 17 (StataCorp. 2023. Stata Statistical Software: Release 17. College Station, TX: StataCorp LLC).

## Results

Table 1 presents the baseline characteristics of the 1091 patients that met the inclusion criteria. Overall, there were more women (66,6%) than men, and the mean age was 42,8 years (SD 10,5). Most patients worked in the public sector (56,5%). Regarding benefits, 59,6% of the patients were on SA and had a mean duration of 103,9 days (SD 90,3) for their current SA spell. There were 11,3% receiving WAA. Most patients reported suffering from musculoskeletal problems (76,1%), and 74,3% of the patients met the criteria for case level symptoms of anxiety and depression. There were no statistically significant differences between the intervention- and the control group with respect to demographic- or health characteristics. Information on education and work sector was not available for the control group.

**Table 1:** Baseline characteristics

	Total		Intervention		Control	
Variable	n		n		n	
Demographic characteristics						
Gender	1091		558		533	
Men	364	33.4%	173	31.0%	191	35.8%
Women	727	66.6%	385	69.0%	342	64.2%
Age	1091	42.8 (10.5)	558	42.7 (10.3)	533	42.8 (10.7)
Education^a^			548			
Primary School			48	8.9%		
High School			212	38.7%		
Higher education up to 3 years			147	26.8%		
Higher education over 4 years			141	25.7%		
Work sector^a^			524			
Public			296	56.5%		
Private			228	43.5%		
Work status	1091					
Work assessment allowance	123	11.3%	65	11.6%	58	10.9%
Full sickness absence	461	42.3%	242	43.4%	226	42.4%
Graded sickness absence	189	17.3%	90	16.1%	101	18.9%
Work/other	308	28.2%	160	28.7%	148	27.8%
Duration of current sickness absence spell (days)	661	105.7 (92.7)	332	109.4 (95.6)	329	101.9 (89.6)
Health characteristics						
Musculoskeletal problems	1083		554		529	
Not at all	60	5.5%	33	5.9%	27	5.1%
No, to a small extent	81	7.5%	43	7.7%	38	7.1%
A little	118	10.9%	57	10.2%	61	11.4%
Yes, to some extent	330	30.5%	159	28.5%	171	32.1%
Yes, to a large extent	494	45.6%	262	47.0%	232	43.5%
Hopkins symptom checklist-10 sum (1–4)^b^	1063	2.3 (0.6)	548	2.2 (0.6)	525	2.3 (0.6)
Hopkins symptom checklist 10 ≥ 1,85	762	71.7%	383	70.5%	379	72.9%

^a^: questions posed to intervention group only

^b^: calculated for complete cases

Table 2 provides the patients responses on MBW factors among the intervention group. In general, patients expressed greater motivation for work than benefits. The mean score for the work environment questions indicate that the patients experience time pressure and constant thoughts about their job, but also that they experience sufficient respect and prestige (ERI). Patients also report high demands in their work, but at the same time they experience control and social support (DCS). Except for not having much energy left for home or family responsibilities after work, patients did not experience substantial work-home interference (WHI). Regarding bullying, 11,3% of patients report bullying as a problem at the workplace. There were 13,0% of patients reporting problematic exposure to noise, pollution, solvents, or chemicals at their workplace, and 26,1% had jobs which included unfavourable working positions.

**Table 2:** Motivation for work, work environment, and barriers for return to work in the intervention group at baseline

Variable	Valid responses, n	%	Mean	SD
Motivation for work				
Believe to be in 12 months (1: do not believe – 5: do believe)				
Work, present job full time	494		3.2	1.7
Work, present job part time	442		2.1	1.4
Work, other job	450		2.6	1.5
Graded sickness absence	429		1.5	1.0
Full sickness absence	429		1.4	1.0
Long term benefits	447		1.9	1.4
Wish to be in 24 months (1: do not wish – 5: do wish)				
Work, present job full time	506		3.7	1.6
Work, present job part time	435		2.2	1.6
Work, other job	457		3.0	1.6
Graded sickness absence	435		1.4	1.0
Full sickness absence	436		1.3	0.9
Long term benefits	443		1.7	1.3
Work environment				
Effort reward imbalance: Time pressure (1: strongly disagree – 4: strongly agree)	523		2.7	1.0
Effort reward imbalance: Sufficient respect and prestige (1: strongly disagree – 4: strongly agree)	518		2.7	0.8
Effort reward imbalance: Constant thoughts about work (1: strongly disagree – 4: strongly agree)	518		2.9	0.9
Work demands: Work demands too much effort (1: no, almost never – 4: yes, often)	519		3.1	0.8
Work control: Choice in deciding how work can be done (1: no, almost never – 4: yes, often)	521		3.1	0.8
Work social support: My colleagues are there for me (1: strongly disagree – 4: strongly agree)	520		3.0	0.7
Work-home interference: Job demands affect time with family (1: never – 4: almost always)	517		1.8	0.8
Work-home interference: Little energy left for home (1: never – 4: almost always)	522		3.0	0.9
Work-home interference: Family demands limit additional job responsibilities (1: never – 4: almost always)	514		1.7	0.9
Work-home interference: Family demands distract work focus (1: never – 4: almost always)	519		1.5	
Is bullying a problem at your workplace?	522			
No	463	88.7		
Yes	46	8.8		
Sometimes	13	2.5		
Exposure to noise, pollution, solvents, or chemicals	516			
No	240	46.5		
Yes, but it`s not a problem	209	40.5		
Yes, and it is a problem	67	13.0		
Exposed to unfavourable working positions	517			
No	182	35.2		
Yes, but it`s not a problem	200	38.7		
Yes, and it is a problem	135	26.1		
**Barriers for return to work **^**a**^	304			
At least 1 barrier with max score	256	84.2		
No barriers with max score	48	15.8		

^a^: only posed to patients reporting to be sick-listed

Table 3 shows the effect of the Nudge on the average accumulated days of benefits, SA or work/other (including education or unemployed) during the 365-day observation window for the 1091 included individuals. Both groups had a similar average of days on DP (difference of 0,3 days), WAA (difference of 6,6 days), FSA (difference of 2,7 days), GSA (difference of 0,4 days), and work/other (difference of 8,5 days). There were no statistically significant differences between the intervention group and the control group for any of the specified outcomes using an independent sample t-test. A robustness check of findings using a non-parametric test in case of non-normal distribution provided the same result.

**Table 3:** Days on disability benefit, work assessment allowance, full sickness absence, graded sickness absence, or work/other during 12 months of follow-up in the intervention and control group

	Intervention (n = 558)			Control (n = 533)			Effects (mean comparison)		
	Mean	SD	CI	Mean	SD	CI	Mean diff	CI	P for diff
Days of DP	1.8	20.0	0.2, 3.5	2.1	21.9	0.3, 4.0	-0.3	−2.8, 2.2	0.81
Days of WAA	78.6	130.0	67.8, 89.4	72.0	126.7	61.2, 82.7	6.6	−8.6, 21.9	0.39
Days of FSA	85.0	105.1	76.2, 93.7	82.3	105.1	73.4, 91.3	2.7	−9.9, 15.1	0.68
Days of GSA	36.5	80.6	29.8, 43.2	36.9	78.7	30.2, 43.6	−0.4	−9.9, 9.0	0.93
Days of work/other	163.2	138.5	151.7, 174.7	171.7	137.0	160.0, 183.5	−8.5	−25.0, 7.9	0.31
Sum	365			365					

*DP* disability pension, *WAA* work assessment allowance, *FSA* full sickness absence, *GSA* graded sickness absence

Furthermore, as shown in Supplementary Fig. 1, we observed no pattern of more work and less SA or benefits in the intervention group during 1-year follow-up. Although the relative distribution of accumulated days per work status/benefit per month was slightly different between randomisation groups, the difference was not statistically significant at any month during follow-up except for days on GSA in month 11, which was statistically significantly lower for the control group (mean diff 0.7 days, p = 0.049).

## Discussion

In this multicentre RCT we aimed to nudge patients and their clinicians towards an increased focus on MBW factors, with the intention to improve work outcomes in a group of patients with common mental- and/or musculoskeletal disorders. The nudge did not have the intended effect. During the 12-month follow-up window we observed no significant differences in accumulated working days or accumulated days on any form of benefit between the intervention- and control group.

To the best of our knowledge, this study is the first to test whether nudging both patients and clinicians towards an increased attention to MBW factors in a work-focussed specialist healthcare setting has an effect on work outcomes or benefits. Studies have shown that questionnaires can influence RTW [51], and there are also evidence to support that posing questions on a particular phenomenon to patients can affect both their perception and awareness [52]. The nudge was meant to inspire patient-clinician dialogue on factors concerning MBW where relevant, and although there is theoretical support for the mechanism of the nudge, it did not have the hypothesised effect on work or benefits. While nudging through a questionnaire offers a possible framework for enhancing focus on work-related factors, the mechanisms underlying successful RTW can be complex, with various factors potentially influencing the RTW process [11, 12]. Some are intrinsic and specific to the individual, while others are external and related to workplace conditions, labour market conditions, or compensation systems [20, 53]. In a therapeutic setting, it may be beyond the scope or power of a clinician to change or influence structural issues even if they were identified through a questionnaire. However, while integrating work-focus in a treatment setting may have a positive impact on RTW outcomes [4, 15, 54], our result aligns with previous literature where there is no conclusive evidence as to which type of work-focussed intervention is best with respect to RTW [16, 18]. A recent RCT conducted in a Norwegian primary care setting tested a work-focussed CBT communication tool for GPs and reported profound effects on SA within a shorter follow-up window [55]. This indicates a potential for including work-focus in the therapeutical management of patients at risk of SA, but in our trial the clinicians delivering the intervention already had experience in work-focussed therapy. This means it may have been difficult to exert more focus than what was already integrated in the clinics. In general, including variants of CBT-based interventions have shown promising results for RTW outcomes for both MSD and CMD [17]. The nudge in the present study was perhaps not comprehensive enough, or the potential for work focus may already have been exhausted in this particular setting. In a previous RCT conducted in a specialist physical rehabilitation clinic, the authors found no difference in RTW during 24 months of follow-up between a group receiving a more comprehensive focus on working conditions, compared to a group receiving brief intervention [56], although this study did not attempt to nudge clinicians or participants. In summary this nudge of both clinicians working in a specialised work-focussed outpatient clinic, and their patients, had no effect on work- or benefit outcomes during 12 months of follow-up. In the supplementary analyses of monthly differences in work or benefits during follow-up we did observe a significant difference in days of GSA in month 11, where the control group had significantly fewer days of GSA than the intervention group (p = 0.049), but we consider this a chance finding of low statistical significance and no practical- or clinical importance.

The findings add to a growing body of evidence on work-focussed interventions provided in clinical settings by testing an innovative and potentially cost-effective approach like nudging through a survey. Given the effects of SA on both the individual and societal level, there is a need for innovative approaches to clinical management of patients with CMD or MSD. Although the intervention did not have an effect on work outcomes, it may still have influenced clinicians` focus and efficiency. Future studies should explore whether similar work-focussed approaches, nudges, and interventions have an effect when applied in other therapeutic settings such as primary care. On a final note, the control condition in the present study reflects ordinary treatment at the HIA clinics, and whether this approach is superior to primary care management is currently being tested in an ongoing trial with results anticipated in late 2026 [45].

## Strengths and Limitations

The study`s strengths include its multicentre randomised controlled design, the relatively large sample size and the use of comprehensive and complete registry data on all benefits over 12 months of follow-up. The collaboration between researchers and clinicians ensured a naturalistic setting. The sickness certifications were prescribed by GP`s, meaning our main outcome assessors were not part of the study. The study was adequately powered, thus avoiding type II error. Statistical power and randomisation ensured that the intervention- and the control group did not differ significantly according to any of the included variables. However, as data on education was not available for the control group, we were unable to assess whether the groups differed significantly on this particular variable. The trial was conducted in a clinic where clinicians already addressed work factors, and this may have reduced the potential impact of the nudge. Clinicians were also involved in developing the questionnaire and have training in how to handle the different MBW factors. Because the study randomised patients, not clinicians, there may have been spillover which primed clinicians to focus on MBW factors inadvertently also for the control group, and this may have influenced results. Finally, given the highly specialised work-focussed setting, the findings and content of the nudge may not be generalisable to other settings.

## Conclusion

A nudge intervention aimed at increasing focus on motivation for work, barriers for return to work and work environment factors did not significantly improve work outcomes compared to usual care in a specialist health care outpatient clinic. The result suggests that an increased work-focus does not enhance work outcomes or reduce sickness absence or benefits in a setting where clinicians already address these factors, albeit in a less structured manner.

## Supplementary Information

Below is the link to the electronic supplementary material.

Supplementary file1 (TIFF 33217 KB)

## Data Availability

The data collected in this study are managed by Nordlandssykehuset HF. Availability of the data collected as part of the study are restricted to personnel named in ethics approval, and the patient consent does not cover sharing data with non-Norwegian researchers. The registry data which determine the outcomes of the study have been delivered by the Norwegian Labour and Welfare Administration (NAV), which may grant data to research purposes upon application and provided a Norwegian ethics approval and a data protection impact assessment (DPIA) at a Norwegian research institution.
